# How to access and exploit natural resources sustainably: petroleum biotechnology

**DOI:** 10.1111/1751-7915.12793

**Published:** 2017-08-03

**Authors:** Angela Sherry, Luiza Andrade, Anne Velenturf, Beate Christgen, Neil D. Gray, Ian M. Head

**Affiliations:** ^1^ School of Natural and Environmental Sciences Newcastle University Newcastle NE1 7RU UK; ^2^ School of Civil Engineering University of Leeds Leeds LS2 9JT UK

## Abstract

As we transition from fossil fuel reliance to a new energy future, innovative microbial biotechnologies may offer new routes to maximize recovery from conventional and unconventional energy assets; as well as contributing to reduced emission pathways and new technologies for carbon capture and utilization. Here we discuss the role of microbiology in petroleum biotechnologies in relation to addressing UN Sustainable Development Goal 12 (ensure sustainable consumption and production patterns), with a focus on microbially‐mediated energy recovery from unconventionals (heavy oil to methane), shale gas and fracking, bioelectrochemical systems for the production of electricity from fossil fuel resources, and innovations in synthetic biology. Furthermore, using wastes to support a more sustainable approach to fossil fuel extraction processes is considered as we undertake the move towards a more circular global economy.

## Abstract 2

How to access and exploit natural resources sustainably: petroleum biotechnology.

Current production and consumption patterns drive growing resource scarcity while, paradoxically, we let these resources dissipate into wastes (Velenturf, A.P.M. and Purnell, P. unpublished data.). Due to the combination of growth in population size and, more importantly, per capita income, resource extraction has accelerated since the start of the century (Schandl *et al*., 2016). The extraction and use of resources, to meet growing demand for food, water, timber, fibre and fuel, have altered the environment to the extent that several planetary boundaries (that indicate the safe operating space for humanity) have been crossed (MEA, 2005; Rockström *et al*., 2009; Steffen *et al*., 2015). This includes the planetary boundary regarding climate change, with adverse impacts on the daily lives of billions of people (IPCC, 2014; UNEP, 2015). The emission of greenhouse gases which contribute to climate change is mainly driven by the burning of fossil fuels and land‐use changes. More than half of industrial carbon emissions are associated with the processing of primary materials (Allwood *et al*., 2012). Adopting a circular economy that extends and recycles these resources within our economy, instead of letting them go to waste, can significantly contribute to carbon emission reductions. However, the circularity of the current global economy is estimated to be limited: Haas *et al*. (2015) found that of the 62Gt materials processed every year, only 13Gt entered the waste stream of which 4Gt was recycled. This is partly because 44% of processed resources are used as energy carriers, which contain energy but do not produce energy (e.g. hydrogen, petroleum, coal, wood) and thus are not available for material recycling. In 2005, 12Gt fossil energy carriers were extracted of which 98% were combusted for energy conversion; seeing as this process is irreversible, it strengthens the demand for recycling of products with high embodied carbon values while it also evidences the need for a new energy future. This includes low‐energy resource recovery solutions (e.g. Gomes *et al*., 2016; Sapsford *et al*., 2016), energy efficiency improvements and the uptake of renewables (IEA, 2016). While renewable markets are forecast to steadily grow, fossil fuels will continue to be part of the energy system for the next decades despite environmental concerns. Energy demand is forecast to grow in non‐OECD countries by 71% from 2012 to 2040. In contrast, in the more mature energy‐consuming and slower‐growing OECD economies, total energy use rises by only 18% from 2012 to 2040 (US EIA, 2016).

On 1 January 2016, the 17 Sustainable Development Goals (SDGs) of the 2030 Agenda for Sustainable Development officially came into force (UN, 2015). Over the next 15 years, with these new Goals that universally apply to all, countries have committed to mobilize efforts to end all forms of poverty, fight inequalities and tackle climate change, while ensuring that no one is left behind. The SDGs are not legally binding, but governments are expected to establish national frameworks for their achievement and to follow up and review progress in implementing the goals. This will require high quality and accessible data arising from new and often innovative research. Over the next half‐century, governments, energy users and producers will have to focus on four issues simultaneously: meet the soaring demand for energy; keep supplies secure; reduce energy's environmental and social impacts; and combat poverty in a fast‐growing global population (UN, 2015). The UN Sustainable Development Goals should not be considered in isolation, as the impact against one goal will invariably impact against other goals. Take, for example, the correlation between energy (Goal 7 and Goal 12) and poverty (Goal 1) using China as an example (Figure 1). It is clear that energy use, income and economic growth are directly coupled, with energy demand exploding above USD 2000/capita GDP, as industrialization and personal mobility increase (Figure 1). Interactions clearly also exist between energy (Goal 7) and climate change action (Goal 13) (IEA, 2016). Resource recovery from waste connects further decarbonization to increasing sustainable, circular systems of production and consumption to limit energy input required for the mining, processing and end‐of‐life treatment of materials, components and products – relevant to achieving almost all SDGs and not least Goal 12 (ensure sustainable consumption and production patterns).

**Figure 1:**
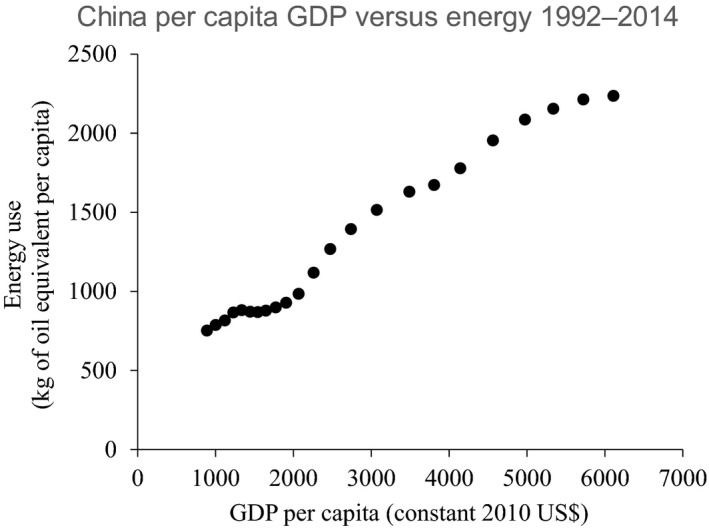
Relationship between energy use per capita and GDP per capita in China (1992‐2014). Data Source – United Nations Statistics Division, https://unstats.un.org/unsd/snaama/dnlList.asp

When transitioning from fossil fuel reliance to a lower‐carbon energy future, it remains to be seen how global and regional government policies will dictate the direction of future energy landscapes as they try to address the reality of population growth, the development necessary for the eradication of poverty and the need to limit carbon emissions. Growth of global energy consumption is increasingly being met by non‐fossil fuels. By 2035, the fuel mix will continue to transition, although oil and gas, together with coal, will remain the dominant sources of energy. Gas will be the fastest growing fuel (1.6% p.a.), with its share in primary energy increasing as it overtakes coal to be the second‐largest fuel source after oil, by 2035. Renewable energy (wind, solar, geothermal, biomass and biofuels) will be the fastest growing source of energy (7.1% p.a.), with its share in primary energy increasing to 10% by 2035, up from 3% in 2015. Renewables, together with nuclear and hydroelectric power, will account for half of the growth in energy supplies over the next 20 years (BP, 2017).

Microbiology impacts many aspects of petroleum exploration and production, and microbial biotechnologies are therefore likely to influence and contribute to the transition from fossil fuels to renewable energy by offering reduced emissions pathways to fossil energy recovery and potential technologies for carbon capture and utilization. There is a growing inventory of petroleum reservoir microbial community data facilitated by the wide and low‐cost availability of high‐throughput sequencing technologies (Caporaso *et al*., 2012). This is furnishing unprecedented information on microbial communities based on the analysis of both 16S rRNA genes and other marker genes as well as metagenomic analysis. While many contemporary studies of petroleum reservoir microbiota are to a degree descriptive and on occasion illustrate some interesting correlations, to date there has been little systematic synthesis of such data to identify the factors that dictate the organisms that thrive under any particular set of reservoir conditions. More adept and sophisticated analyses of these data will first identify robust correlations and ultimately use these to develop testable hypotheses that will lead to a better understanding of how we can manage microbial populations in petroleum reservoirs to facilitate, for example, more reliable microbially mediated energy recovery as outlined below.

## Unconventionals – heavy oil to methane

Unconventional fossil fuels encompass heavy oil and oil sands, shale oil and gas, coal bed methane (CBM) and methane hydrates. There is a potential microbial dimension to the recovery of most of these assets. Unconventional sources of fossil fuels are typically more expensive to produce than conventional reserves, which makes their exploitation highly sensitive to oil price fluctuations. Generally, unconventional reserves are extracted using physical processes (e.g. hydraulic fracturing for CBM or thermal processing such as steam assisted gravity drainage – SAGD – for viscous heavy oils). Thermal processes in particular are costly due to the energy input required to recover the fossil fuels and for subsequent processing to facilitate transport and refining. However, indigenous microbial consortia are able to convert residual oil to methane *in situ* in petroleum reservoirs. The first reports of the capability of methanogenic consortia to convert alkanes to methane (Zengler *et al*., 1999) prompted the possibility that this process could be harnessed for enhanced recovery of energy from residual oil in reservoirs where conventional secondary and tertiary recovery was no longer feasible (Parkes, 1999). Subsequent demonstrations that methane production from crude oil as a substrate provided further credibility to this notion (Townsend *et al*., 2003; Gieg *et al*., 2008; Jones *et al*., 2008). There are several attractive features of this approach to fossil energy recovery. Methane is ultimately the energy vector produced by the process and it offers reduced CO_2_ emissions per unit of energy generated compared with oil and gas. Moreover, methanogenic degradation of oil produces both CO_2_ and methane, which have different physical and chemical properties, there is potential to recover the methane while retaining the CO_2_ in the subsurface, for example, by maintaining slightly alkaline conditions, reducing the net emissions of fossil carbon for the process. However, attempts to take this technology from the laboratory to practical application (Larter *et al*., 2005; Pfeiffer *et al*., 2008) showed that rates of microbial methane generation from heavy oil and oil sands are 1 to 2 orders of magnitude lower than the rates from light oil (up to 72 lmol/dm3/day, Wang *et al*., 2012). Consequently, it seems that oil conversion into methane for enhanced energy recovery, from heavy oil at least, may not be economically feasible.

## Shale gas and fracking

Shale gas represents one‐third of natural gas energy resources on the planet (Daly *et al*., 2016), and it is estimated that some countries, like the USA, will be highly dependent on it for energy recovery over the next twenty years (US EIA, 2013). Fracking or hydraulic fracturing uses high‐pressure injection of water and other chemicals (e.g. surfactants, solvents, biocides) to release shale gas and hydrocarbon compounds trapped in tiny pore spaces by creating a network of artificial fractures in the source rock. A wide range of environmental impacts associated with fracking operations have been studied including contamination of surface and groundwater, methane emissions, air pollution, migration of gases, hydraulic fracturing chemicals and radionuclides to the surface, the potential mishandling of solid waste, drill cuttings, increased seismicity, effects on human health, with potentially critical effects on diverse ecosystem components (Meng, 2017 and references therein).

Relatively, little is known about the role of microbiology in the fracking industry. Native microbial communities are unlikely to exist in the non‐fractured matrix of shales (Head and Gray, 2016); however, it is possible that native species inhabit the natural fractures. Several studies have observed that hydraulic fracturing creates favourable conditions for microbial growth in shales, despite the use of biocides. Predominantly halotolerant and methanogenic microbial communities have been identified, which play a role in methylamine cycling, methanogenesis, sulfur cycling and fermentation of some of the injected chemical additives (Cluff *et al*., 2014; Daly *et al*., 2016; Liang *et al*., 2016). Recently, in a large metagenomics study of produced fluids from the Marcellus Shale, Pennsylvania, a new genus was identified, *Candidatus* Frackibacter (Daly *et al*., 2016).

The survival and acclimation of microbes can also have negative implications for gas recovery and infrastructure. For example, a recent study in the Barnett Shale, Texas, showed a dominance of *Halanaerobium* associated with unconventional shale gas extraction, which proliferated and produced sulfide and acetate from the metabolism of polysaccharides (guar gum) used in the hydraulic fracturing fluids. Such microbial metabolisms can lead to gas souring and corrosion of carbon‐steel equipment in the industry. The study subsequently investigated biocides to inhibit the growth and metabolic activity of the microorganisms, in order to mitigate the deleterious effects of the sulfide (Liang *et al*., 2016). Investigations into microbe‐mineral interactions within the fractures may be a future strategy to manipulate the porosity and permeability of the shale matrix through microbially mediated mineral precipitation or dissolution as a means to exploit the shale gas resource (Head and Gray, 2016).

## Bioelectrochemical systems

Economic, environmental, social and political changes in the landscape of the oil industry and the development of powerful new technologies to measure and understand complex microbial ecosystems like petroleum reservoirs make the development of microbial technologies for enhanced energy recovery from fossil fuel reserves possible. However, there are still frontier technologies that have been little explored in the context of the microbiology of fossil fuels. As one of these emerging areas, bioelectrochemical technologies have the potential to recover energy directly as electricity while leaving the carbon in the ground.

Greater knowledge of electron transfer processes, such as direct intracellular electron transfer (DIET) (Rotaru *et al*., 2014), is warranted. The observation that the ability to participate in syntrophic relationships based on DIET is linked to the ability to respire solid‐phase electron acceptors, such as electrodes (Rotaru *et al*., 2015), demonstrates a larger than anticipated potential of the microbial world to couple oxidation of organic matter to electrode respiration and hence electricity generation. It has been speculated that this could form the basis for a large‐scale bioelectrochemical system for harvesting electricity directly from petroleum reservoirs (Head *et al*., 2014). Anodes would be inserted into the organic carbon‐rich reservoir sediments or the oil‐water transition zone, microbial oxidation of crude oil components would then be linked to anode‐reduction with the anode connected to some form of air cathode at the surface. This could potentially generate energy while leaving the oxidized carbon in the reservoir. Similarly, oil spills could be remediated concomitantly with electricity generation using a bioelectrochemical approach referred to as the ‘Oil‐Spill Snorkel’ (Viggi *et al*., 2015). In this system, a conductive electrode (the ‘Snorkel’) connects the contaminated anoxic zone and the overlying oxygen containing water (oxic zone) to stimulate the oxidative biodegradation of hydrocarbons in sediments. This mimics the spatial separation of oxidation and reduction processes observed in ‘cable bacteria’ (conductive and long multicellular filamentous bacteria with electron transporting structures inside a common periplasm) (Nielsen and Risgaard‐Petersen, 2015) which circumvents the conventional cascade of redox processes and may drive oxygen consumption in sediment/water zones.

## Synthetic Biology in alternative energy production

Recent technological advances in synthetic biology likely mean it will play a role in energy recovery from fossil fuels or in energy production in the near future. A recent innovative synthetic biology solution with the potential for enhanced energy production is SimCells (Huang, 2013). These are minicells with designed circuits in plasmids which instruct cells to perform specific functions, such as the production of alkanes from CO_2_, powered by electricity and light through bio‐electrosynthesis (Huang, 2013). SimCells can be safely used in the environment, as they cannot reproduce themselves. Moreover, the increasing scarcity of fossil fuels has led to the enhanced production of bio‐based fuels as an alternative source of energy. Synthetic biology tools are currently being developed in Yeasts for the increased production of biofuels, particularly bioethanol (reviewed in Madhavan *et al*., 2017).

## Using wastes in support of more sustainable extraction processes

The preceding sections have already indicated the important role that microbial biotechnologies can play in fossil fuel exploration and extraction and that this is becoming increasingly feasible due to improved measurement technologies. Using microbial biotechnologies can be a cost‐effective alternative to the chemical products on the market. For example, Omajali *et al*. (2017) argue that using noble metals on chemical supports as catalysts is expensive, and propose to use nanoparticles on biological supports instead. They produced a catalyst from precious metals using hydrogen and bacterial cells, sourced from road dust, for the *in situ* upgrading of heavy oils. Finding a valuable outlet for this waste stream has economic and environmental benefits, while the use of this catalyst also reduces the energy required in the heavy oil extraction process. These benefits support uptake of the technology in industry, while they can also help making the business case for governments. Clearly continued fossil fuel extraction is controversial given the impacts of climate change (IEA, 2016; Rockström *et al*., 2009), however, since we continue to be dependent on fossil fuels it is important to make the extraction and consumption processes as sustainable as possible. Using wastes to increase sustainability of the extraction process will offer governments opportunity to steer developments in this industry, for example through tax instruments targeting raw materials and/or wastes (Söderholm, 2011).

## Conclusions

Microbial biotechnologies contribute to the fossil fuel industry in a number of ways, by assisting in the exploration and production of fossil fuels, upgrading fuels, bioremediation of spills, and in the control of souring of petroleum reservoirs and the corrosion of infrastructure. Increasing the recovery of energy from depleted petroleum reservoirs in the microbial conversion of residual oil to methane, particularly in combination with CO_2_ sequestration into the subsurface, continues to warrant further investigation. The production of biofuels, such as bioethanol, has increased dramatically in recent years, and this trend is likely to continue with advances in synthetic biology. Increasing the knowledge of the microbial communities involved in hydraulic fracturing operations will provide important information on the biogeochemical processes occurring in the fractured subsurface environment and may help to troubleshoot shale gas production issues. However, the contributions of microbial biotechnologies to the energy industry are not only influenced by technical advances, but also by economic, environmental, social and political changes globally, for example, the price of fossil fuels, global population growth, the development of renewables, concerns about the use of crops for food versus fuel production. To move towards addressing the UN SDGs by 2030, governments, energy users and producers require a new global energy infrastructure to mitigate the effects from global warming on the environment. The developed world drastically needs to cut its own emissions and collaborate with developing countries to expand renewables. To implement this, governments will be required to lay down long‐term policies for a pathway to a lower‐carbon world and encourage a move to a more circular global economy that attempts to extend and recycle scarce resources, instead of letting them go to waste, to ultimately ensure we are able to continue to meet the energy needs of a changing world.

## Conflict of interest

None declared.
